# Building Digital Literacy In The Older Adult North Nashville Community

**DOI:** 10.1093/geroni/igaf122.2796

**Published:** 2025-12-31

**Authors:** Jennifer Kim, Cathy Maxwell

**Affiliations:** Vanderbilt University, Nashville, Tennessee, United States; University of Utah, Salt Lake City, Utah, United States

## Abstract

Digital literacy (DL), the ability to understand and use digital technology, is crucial for older adults’ mental and physical well-being. Using an academic and community partnership model, investigators developed an evidence-based, 8-week, in-person digital literacy program (DLP) for low-income older adults in an African American community to examine effects on participants’ DL abilities. Participants were recruited from two senior centers. The DLP curriculum was based on the Mobile Device Proficiency Questionnaire (MDPQ) subscales (e.g., mobile device basics, communication, date/file creation and storage, internet access, software management). All participants received a personal electronic tablet. Reinforcement of the DLP curriculum and social support was provided through weekly engagement with volunteer student nurses. Pre/post digital literacy was measured using the MDPQ. Forty older adults participated in the program and completed an initial baseline survey on digital literacy tasks. Compared to baseline assessments, statistically significant improvements were noted for 17 tasks, which participants felt were “very easy” or “somewhat easy” to perform. Tasks with the strongest improvements included the ability to find information about local community resources (pre: 9, 25% vs. post: 20, 50%, p<.001) and about hobbies and interests (pre: 11, 27.5% vs. post: 19, 47.5%, p<.001), and the ability to enter events and appointments into a calendar (pre: 6, 15% vs. post: 16, 40%, p<.001). Digital literacy programs for older adults are wise investments for both private and public funding sponsors. The academic and community partnership model is an effective way to support nonprofit organizations that lack resources.

